# “Sugar-Sweetened Beverages” Is an Independent Risk From Pancreatic Cancer: Based on Half a Million Asian Cohort Followed for 25 Years

**DOI:** 10.3389/fonc.2022.835901

**Published:** 2022-04-07

**Authors:** Chien Hua Chen, Min Kuang Tsai, June Han Lee, Ro-Ting Lin, Chung Y. Hsu, Christopher Wen, Xifeng Wu, Ta-Wei Chu, Chi Pang Wen

**Affiliations:** ^1^ Digestive Disease Center, Changbing Show-Chwan Memorial Hospital, Lukang, Taiwan; ^2^ Department of Post-Baccalaureate Medicine, College of Medicine, National Chung Hsing University, Taichung, Taiwan; ^3^ Department of Food Science and Technology, Hungkuang University, Taichung, Taiwan; ^4^ Institue of Population Health Sciences, National Health Research Institutes, Zhunan, Taiwan; ^5^ College of Public Health, China Medical University, Taichung, Taiwan; ^6^ Graduate Institute of Clinical Medical Science, China Medical University, Taichung, Taiwan; ^7^ Long Beach VAMC Hospital, University of Irvine Medical Center, Irvine, CA, United States; ^8^ Center for Biostatistics, Bioinformatics and Big Data, The Second Affiliated Hospital and School of Public Health, Zhejiang University School of Medicine, Hangzhou, China; ^9^ National Institute for Data Science in Health and Medicine, Zhejiang University, Hangzhou, China; ^10^ Department of Obstetrics and Gynecology, Tri-Service General Hospital, National Defense Medical Center, Taipei, Taiwan; ^11^ Taipei MJ Health Screening Center, Taipei, Taiwan

**Keywords:** sugar-sweetened beverages, pancreatic cancer, mortality, incidence, cohort

## Abstract

Although the link between sugar-sweetened beverages (SSB) and pancreatic cancer has been suggested for its insulin-stimulating connection, most epidemiological studies showed inconclusive relationship. Whether the result was limited by sample size is explored. This prospective study followed 491,929 adults, consisting of 235,427 men and 256,502 women (mean age: 39.9, standard deviation: 13.2), from a health surveillance program and there were 523 pancreatic cancer deaths between 1994 and 2017. The individual identification numbers of the cohort were matched with the National Death file for mortality, and Cox models were used to assess the risk. The amount of SSB intake was recorded based on the average consumption in the month before interview by a structured questionnaire. We classified the amount of SSB intake into 4 categories: 0–<0.5 serving/day, ≥0.5–<1 serving per day, ≥1–<2 servings per day, and ≥2 servings per day. One serving was defined as equivalent to 12 oz and contained 35 g added sugar. We used the age and the variables at cohort enrolment as the reported risks of pancreatic cancers. The cohort was divided into 3 age groups, 20–39, 40–59, and ≥60. We found young people (age <40) had higher prevalence and frequency of sugar-sweetened beverages than the elderly. Those consuming 2 servings/day had a 50% increase in pancreatic cancer mortality (HR = 1.55, 95% CI: 1.08–2.24) for the total cohort, but a 3-fold increase (HR: 3.09, 95% CI: 1.44–6.62) for the young. The risk started at 1 serving every other day, with a dose–response relationship. The association of SSB intake of ≥2 servings/day with pancreatic cancer mortality among the total cohort remained significant after excluding those who smoke or have diabetes (HR: 2.12, 97% CI: 1.26–3.57), are obese (HR: 1.57, 95% CI: 1.08–2.30), have hypertension (HR: 1.90, 95% CI: 1.20–3.00), or excluding who died within 3 years after enrollment (HR: 1.67, 95% CI: 1.15–2.45). Risks remained in the sensitivity analyses, implying its independent nature. We concluded that frequent drinking of SSB increased pancreatic cancer in adults, with highest risk among young people.

## Introduction

The public health significance of pancreatic cancer and its increasing mortality have been largely ignored. Most incidence died within a few years and incidence correlated with mortality. In the United States, a 1.6 and 1.9% increase per year in incidence and mortality of pancreatic cancer, respectively, has been observed (1–4). A trend of rapid increase in incidence has also been noted in Taiwan, with a 3-fold increase in incidence in the recent 2 decades (**Supplementary Figures 1–2**) (5, 6). Pancreatic cancer is now the fourth leading cause of cancer deaths in all age groups and the third leading cause of cancer deaths among people aged ≥40 in the United States (1, 2). Most people are more familiar with colorectal cancer than with pancreatic cancer; in 2021, colorectal cancer cases (149,500) were 2–3 times higher than pancreatic cancer cases (60,430) in the United States, which may contribute to less attention being paid to pancreatic cancer (1). However, the number of pancreatic cancer deaths has been increasing faster than that of colorectal cancer, approximating that of colorectal cancer in 2021 (pancreatic cancer: 48,220 vs. colorectal cancer: 52,980). This implies that the seriousness of pancreatic cancer or the need for its prevention has been under-appreciated. The reduction of pancreatic cancer cases merits the same attention as that devoted to reducing colorectal cancer cases.

Only a few lifestyle risk factors, such as smoking and drinking, have been identified for pancreatic cancer, but the size of increase in incidence due to these lifestyle risks is minimal (7–9). Moreover, smoking and drinking have both been highly prevalent in the United States and Taiwan for decades before the recent concerns regarding the rapidly increasing pancreatic cancer cases. Furthermore, neither smoking nor drinking could account for the strong increasing trend observed in the last 20 years (3, 10).

The overall consumption of sugar-sweetened beverages (SSBs), the main source of added sugars in diets of people, has remained high since 1970s (11–14). Furthermore, a survey conducted between 2013 and 2016 revealed that approximately 83.6% of Taiwanese adults (age: 19–44 years) consumed more than 1 serving/week of SSB, with a mean of 7.8 servings/week (15). Sweetened beverages in Asia also include a popular drink among young people—bubble milk tea—which is a Taiwan specialty drink containing tapioca. The amount of sugar added to this drink can be excessive. Studies have suggested an association between SSBs and pancreatic cancer (16–19), most probably due to SSB-induced rapid increase in blood sugar, which stimulates insulin secretion and cancer cell proliferation (20, 21). A high sugar intake induces hyperinsulinemia, consequently enhancing carcinogenesis by inhibiting apoptosis and downregulating binding protein 1 for the insulin-like growth factor (22). Fructose syrup, commonly added to sweetened beverages, is rapidly absorbed by glucose transporter 5 and easily induces insulin resistance by hampering the insulin signaling pathway (22). Compared with glucose, fructose is more readily utilized by pancreatic cancer cells through the non-oxidative pentose phosphate pathway to increase ribonucleic acid synthesis (23). The endocrine function of the pancreas and the cancer-causing nature of insulin have made the sugar drinks hypothesis plausible; however, evidence from most epidemiological studies addressing this causal association has been inconclusive (24–37).

SSB consumption has been reported to be associated with increased risks of cardiovascular diseases or all-cause mortality, implying the systemic nature of this risk (25, 38). This study evaluated the pancreatic cancer risk in a cohort of approximately half a million individuals attending a self-paid medical screening program. Completed lifestyle questionnaires and blood test results were collected. The cohort was divided into three age groups (20–39, 40–59, and ≥60 years) at enrollment to examine the association between specific cancers and drinks, and the independent nature of the association was assessed by adjusting for, or excluding, all known confounders. Given the paucity of our knowledge in preventing this highly fatal cancer, the assessment and quantification of risks induced by consuming sweetened beverages could be a crucial public health contribution.

## Materials and Methods

### Study Population

This prospective cohort study enrolled 491,929 individuals aged ≥20 years without known cancer history from the four MJ clinics that have been conducting a self-paid medical screening program across Taiwan with well-administered medical records since 1994 (39, 40). The follow-up period ranged from 1994 to 2017 (median period: 15 years; interquartile range: 9–20 years). The participants paid to become members of the MJ Health Management Institution and to undergo physical check-ups. The battery of examinations was completed within a few hours in the morning, and the complete interpretation and multidisciplinary education for individualized counseling was provided before the participants left the clinic in the afternoon. Many participants were willing to undergo repeated examinations as a result of the efficiency and friendliness of MJ.

### Measurements

The participants underwent sequential blood, urine, and pulmonary function tests and also electrocardiography. They also underwent physical examination and a review of medical history. Moreover, the participants completed a self-report structured questionnaire about lifestyle.

### Questionnaire and Lab Data

#### Hypertension and Diabetes

Hypertension was defined as a self-reported history of hypertension, systolic blood pressure of ≥140 mm Hg, diastolic blood pressure of ≥90 mmHg, or use of antihypertensive agents. Diabetes was defined as a self-reported history of diabetes, fasting blood glucose level of ≥126 mg/dl, or use of hypoglycemic agents.

#### Assessing SSBs

The definition of total SSB included sugar-added drinks such as caffeinated or decaffeinated cola, carbonated SSBs, and noncarbonated SSBs (27). In addition, bubble milk tea, a Taiwan handmade specialty drink with high sugar content, was included. Natural fruit juice without added sugar was not classified as an SSB. The questionnaire referred to SSB consumption in the most recent month with four choices of answer to quantify the amount: 0 to <0.5 serving/day, ≥0.5 to <1 serving per day, ≥1 to <2 servings per day, and ≥2 servings per day. One serving was defined as equivalent to 12 oz or 350 ml and contained 150 Kcal or 35 g added sugar (41).

#### Assessing Physical Activity by Exercise Volume

We classified the leisure time physical activity of each individual into five groups: inactive (<3.75 metabolic equivalent task (MET)-h/week or <5 min/day), low activity (3.75–7.49 MET-h/week or approximately 15 min/day), moderate activity (7.50–16.49 MET-h/week or approximately 30 min/day), high activity (16.50–25.49 MET-h/week or approximately 60 min/day), and very high activity (≥25.50 MET-h/week or approximately 90 min/day or more) (40).

### Assessment of Outcome

The individual identification numbers of the cohort were matched with the National Death File for mortality and with the National Cancer Registry for cancer incidence. We coded mortality according to the *International Classification of Diseases*, *Ninth* and *Tenth* revisions, with pancreatic cancer coded as 157 and C25, respectively. We referred to age and variables at cohort enrollment in this study. Written informed consent was provided by each participant, and this study was approved by the Institutional Review Board of China Medical University in Taiwan. All data were encrypted and remained anonymous during the entire study process.

### Statistical Analysis

The association of SSB with pancreatic cancer can be attributed to unhealthy behaviors accompanying SSB intake, such as smoking or alcohol, or to metabolic syndrome resulting from SSB intake (13–15, 22–24, 38, 42, 43). To support the causal relationship between SSB and pancreatic cancer, we performed several sensitivity analyses to validate our findings: 1) to assess the dose–response relationship, we measured the p-value for the trend between SSB consumption and the pancreatic cancer mortality risk; 2) to mitigate the comorbid effect on pancreatic cancer development, we excluded the subgroups with smoking, alcohol consumption, obesity, hypertension, or diabetes; and 3) to avoid participants with reverse causality or incipient cancer before the study, we excluded those who died within 3 years after enrollment. We calculated the hazard ratios (HRs) with 95% confidence intervals of incidence and mortality by using the Cox model.

Univariate analysis was used to assess the possible risk factors of pancreatic cancer. Variables with statistical significance in univariate analysis were considered for multivariate analysis to assess the association between SSB intake and pancreatic cancer risk. For the total cohort, the HR was adjusted for age, sex, education level, smoking status, drinking status, physical activity, body mass index (BMI), hypertension, and diabetes. In the sensitivity analysis, adjustments for potential confounders were unnecessary. For age-classified models, the HR was adjusted for sex, education level, smoking status, drinking status, physical activity, BMI, hypertension, and diabetes. No violation of the proportional hazard assumption was noted in our study. All statistical analyses were performed using SAS 9.4 (SAS Institute Inc., Cary, NC), and a two-tailed *P <*0.05 was considered significant.

## Results

### Population Distribution of SSB Consumption

The study cohort included 491,929 participants, with 235,427 men and 256,502 women (mean age: 39.9 years, standard deviation: 13.2). The follow-up period was between 1994 and 2017 (median follow-up: 15 years). In total, 523 cases of pancreatic cancer deaths and 489 cases of incident pancreatic cancers were observed during a total of 89 million person-years during follow-up. Because the 5-year survival of pancreatic cancer is 10%, mortality data can be expected to be slightly less than incidence. However, mortality data were available for 2 additional years, censoring by 2017, compared with incidence data, censoring by 2015. Therefore, mortality registered in the National Death File might outnumber the incidence registered in the National Cancer Registry in our study. **Table 1** indicates that the population distribution of SSB consumption from 0 to <0.5 serving/day to ≥2 servings/day tended to increase for those in the 20–39 years age group, men, current smokers, regular drinkers, and obese participants. One-fifth (20.6%) of the total cohort and one-fourth (24.7%) of the young population (age <40 years) reported SSB intake of ≥1 serving/day.

**Table 1:** The population distribution of sugar-sweetened beverages consumption.

		Total cohort Number (%)		0–<0.5 serving/day		≥0.5–1 serving/day		≥1–2 servings/day		≥2 servings/day	
		491,929	(100.0)	324,480	(66.0)	65,962	(13.4)	63,950	(13.0)	37,537	(7.6)
Age at enrollment	20–39	288,747	(100.0)	167,845	(58.1)	49,669	(17.2)	44,388	(15.4)	26,845	(9.3)
	40–59	151,609	(100.0)	112,352	(74.1)	13,965	(9.2)	16,390	(10.8)	8,902	(5.9)
	60 or above	51,573	(100.0)	44,283	(85.9)	2,328	(4.5)	3,172	(6.1)	1,790	(3.5)
Gender	Men	235,427	(100.0)	144,789	(61.5)	35,700	(15.2)	31,733	(13.5)	23,205	(9.8)
	Women	256,502	(100.0)	179,691	(70.1)	30,262	(11.8)	32,217	(12.6)	14,332	(5.5)
Education	Middle school or below	99,556	(100.0)	82,988	(83.4)	4,971	(5.0)	7,164	(7.2)	4,433	(4.4)
	High school	103,500	(100.0)	69,279	(66.9)	11,766	(11.4)	13,724	(13.3)	8,731	(8.4)
	Junior college	99,015	(100.0)	60,507	(61.1)	15,420	(15.6)	14,472	(14.6)	8,616	(8.7)
	College or above	182,253	(100.0)	106,325	(58.3)	32,996	(18.1)	27,706	(15.2)	15,226	(8.4)
Smoking status	Non-smoker	342,264	(100.0)	233,436	(68.2)	46,604	(13.6)	42,456	(12.4)	19,768	(5.8)
	Ex-smoker	29,518	(100.0)	19,765	(67.0)	3,813	(12.9)	3,604	(12.2)	2,336	(7.9)
	Current smoker	105,499	(100.0)	59,508	(56.5)	14,501	(13.7)	16,713	(15.8)	14,777	(14.0)
Drinking status	Non-drinker	377,527	(100.0)	246,193	(65.2)	53,457	(14.2)	50,225	(13.3)	27,652	(7.3)
	Occasional drinker	50,609	(100.0)	33,221	(65.6)	6,424	(12.7)	6,354	(12.6)	4,610	(9.1)
	Regular drinker	39,684	(100.0)	26,935	(67.9)	3,971	(10.0)	4,763	(12.0)	4,015	(10.1)
Physical activity	Inactive	240,751	(100.0)	152,795	(63.5)	33,062	(13.7)	33,498	(13.9)	21,396	(8.9)
	Low	125,316	(100.0)	83,893	(66.9)	17,833	(14.2)	15,829	(12.7)	7,761	(6.2)
	Medium	71,791	(100.0)	49,550	(69.0)	9,203	(12.8)	8,489	(11.9)	4,549	(6.3)
	High	26,868	(100.0)	19,356	(72.0)	2,833	(10.6)	2,854	(10.6)	1,825	(6.8)
	Very high	16,034	(100.0)	10,928	(68.2)	1,832	(11.4)	1,856	(11.6)	1,418	(8.8)
Body mass index	<18.5	43,336	(100.0)	27,095	(62.5)	6,600	(15.3)	6,331	(14.6)	3,310	(7.6)
	18.5–24	315,249	(100.0)	208,336	(66.0)	42,456	(13.5)	41,261	(13.1)	23,196	(7.4)
	25–29	112,093	(100.0)	75,230	(67.1)	14,028	(12.5)	13,702	(12.2)	9,133	(8.2)
	≧30	21,061	(100.0)	13,693	(65.0)	2,860	(13.6)	2,624	(12.5)	1,884	(8.9)
Hypertension	No	404,722	(100.0)	257,913	(63.7)	58,276	(14.4)	55,992	(13.9)	32,541	(8.0)
	Yes	87,207	(100.0)	66,567	(76.3)	7,686	(8.8)	7,958	(9.2)	4,996	(5.7)
Diabetes	No	467,429	(100.0)	304,929	(65.2)	64,195	(13.7)	62,116	(13.4)	36,189	(7.7)
	Yes	24,500	(100.0)	19,551	(76.7)	1,767	(7.3)	1,834	(7.5)	1,348	(5.5)
CKD	No	424,558	(100.0)	276,819	(65.2)	58,681	(13.8)	56,261	(13.3)	32,797	(7.7)
	Yes	44,255	(100.0)	33,100	(74.8)	3,969	(8.9)	4,149	(9.4)	3,038	(6.9)

We referred to the age and the variables at cohort enrolment in this study; Body mass index, kg/m^2^; CKD, Chronic kidney disease stage 1–5.

### Risk Factors Associated With Pancreatic Cancer

Risk factors found from the Cox model for pancreatic cancer, either incidence or mortality, were age, male sex, SSB consumption, smoking, alcohol drinking, overweight or obesity (BMI ≥25 or 30 kg/m^2^), hypertension, and diabetes (**Table 2**).

**Table 2:** Univariate analysis of the possible risk factors for pancreatic cancer mortality and incidence among the total cohort.

		Number of subjects	Pancreatic cancer mortality			Pancreatic cancer incidence		
			n	HR	95% CI	n	HR	95% CI
Total		491,929	523			489		
Sugar drinks	0–<1 serving/week	191,076	342	1.00		306	1.00	
	>1 serving/week–<0.5 serving/day	133,404	80	0.86	0.67–1.11	80	0.85	0.66–1.10
	≥0.5–<1 serving/day	65,962	23	0.70	0.46–1.08	26	0.76	0.50–1.14
	≥1–<2 servings/day	63,950	39	1.01	0.72-1.41	38	0.96	0.68–1.35
	≥2 servings/day	37,537	39	1.55	1.11–2.18	39	1.51	1.07–2.13
Age at enrollment	20–39	288,747	48	1.00		58	1.00	
	40–59	151,609	207	7.83	5.72–10.72	193	6.47	4.80–8.71
	60 or above	51,573	268	31.49	23.16–42.82	238	25.53	19.07–34.16
Gender	Men	235,427	277	1.30	1.10–1.54	256	1.25	1.04–1.49
	Women	256,502	246	1.00		233	1.00	
Education	Middle school or below	99,556	306	1.47	1.07–2.01	264	1.33	0.97–1.82
	High school	103,500	90	1.36	0.97–1.91	95	1.43	1.02–1.99
	Junior college	99,015	64	1.47	1.03–2.11	67	1.48	1.04–2.11
	College or above	182,253	55	1.00		58	1.00	
Smoking status	Non-smoker	342,264	330	1.00		317	1.00	
	Ex-smoker	29,518	36	0.74	0.51–1.07	38	0.88	0.62–1.27
	Current smoker	105,499	129	1.27	1.01–1.61	112	1.16	0.91–1.49
Drinking status	Non-drinker	377,527	336	1.00		332	1.00	
	Occasional drinker	50,609	66	1.31	0.99–1.72	52	1.09	0.80–1.47
	Regular drinker	39,684	81	1.39	1.07–1.81	73	1.30	0.99–1.71
Physical activity	Inactive	240,751	237	1.00		216	1.00	
	Low	125,316	105	1.02	0.81–1.28	91	0.97	0.76–1.24
	Medium	71,791	85	0.79	0.62–1.02	86	0.89	0.69–1.15
	High	26,868	55	0.86	0.64–1.16	56	1.03	0.76–1.39
	Very high	16,034	29	0.89	0.60–1.32	28	1.01	0.68–1.51
Body mass index	<18.5	43,336	19	0.99	0.62–1.57	16	0.84	0.51–1.39
	18.5–24	315,249	286	1.00		274	1.00	
	25–29	112,093	187	1.27	1.05–1.52	170	1.24	1.02–1.50
	≧30	21,061	31	1.48	1.02–2.15	29	1.42	0.96–2.09
Hypertension	No	404,722	269	1.00		257	1.00	
	Yes	87,207	254	1.30	1.07–1.56	232	1.33	1.09–1.62
Diabetes	No	467,429	423	1.00		409	1.00	
	Yes	24,500	100	1.87	1.49–2.34	80	1.57	1.22–2.00

We referred to the age and the variables at cohort enrolment in this study; Body mass index, kg/m^2^.

### SSB Consumption and Pancreatic Cancer Risks

The total cohort exhibited a 50% increase in pancreatic cancer mortality (HR = 1.55, 95% CI: 1.08–2.24) and incidence (HR = 1.55, 95% CI: 1.08–2.23) for those consuming 2 servings/day of SSB, with a dose–response relationship observed when the intake was greater than 1 serving every other day (**Table 3** and **Supplementary Table 1**).

**Table 3:** The mortality risks of pancreatic cancer by levels of consumed sugar-sweetened beverages.

		Number of subjects	Total deaths of pancreatic cancer	0–<0.5 serving/day		≥0.5–<1 serving/day			≥1–<2 servings/day			≥2 servings/day			≥ 1 serving/day			P for trend*	P for trend**
	Age (mean ± SD)			N of death	HR	N of death	HR	95% CI	N of death	HR	95% CI	N of death	HR	95% CI	N of death	HR	95% CI		
Total cohort	39.9 ± 13.2	491,929	523	422	Ref	23	0.64	0.39–1.05	39	0.95	0.65–1.40	78	1.20	0.91-1.58	39	1.20	1.08-2.24	0.172	0.003
Age at enrollment																			
20–39	30.6 ± 4.8	281,747	48	27	Ref	4	0.89	0.31–2.61	7	1.48	0.60–3.67	17	2.18	1.13-4.21	10	2.18	1.44-6.62	0.008	0.029
40–59	48.5 ± 5.9	151,609	207	167	Ref	9	0.54	0.24–1.23	13	0.69	0.35–1.36	31	0.99	0.62-1.57	18	0.99	0.80-2.54	0.948	0.064
≥60	66.5 ± 5.6	51,573	268	228	Ref	10	0.80	0.38–1.70	19	1.19	0.69–2.06	30	1.24	0.81-1.91	11	1.24	0.69-2.50	0.390	0.285

We referred to the age and the variables at cohort enrolment in this study.

Univariate analysis was used to assess the possible risk factors of pancreatic cancer. Those variables with statistical significance were considered for multivariate analysis for the association between SSB intake and the risk of pancreatic cancer.

For total cohort, HR was adjusted for categories of age, gender, education levels, smoking status, drinking status, physical activity, body mass index, hypertension, and diabetes.

For age-classified models, HR was adjusted for gender, education levels, smoking status, drinking status, physical activity, body mass index, hypertension, and diabetes.

SD, standard deviation.

*Trend starts from 0 to <0.5 serving/day.

**Trend starts from ≥0.5 to <1 serving/day.

### Sensitivity Analysis for the Relation of SSB Consumption With Pancreatic Cancer

>**Table 4** presents the results of sensitivity analysis for the relation of SSB consumption with pancreatic cancer mortality based on SSB consumption levels. The association of SSB intake of ≥2 servings/day with pancreatic cancer mortality among the total cohort was significant after excluding those who smoke or have diabetes (HR: 2.12, 97% CI: 1.26–3.57), are obese (HR: 1.57, 95% CI: 1.08–2.30), have hypertension (HR: 1.90, 95% CI: 1.20–3.00), and who died within 3 years after enrollment (HR: 1.67, 95% CI: 1.15–2.45). The sensitivity analysis results for the young group (aged 20–39) are presented in **Supplementary Table 2**. Because relatively few pancreatic cancer deaths were noted in the total cohort, age and variables at enrollment were adjusted for these models in **Tables 3**, **4** (44).

**Table 4:** Sensitivity analyses for the mortality risks of pancreatic cancer by levels of consumed sugar-sweetened beverages.

	0–<0.5 serving/day		≥0.5–<1 serving/day			≥1–<2 servings/day			≥1 serving/day			≥2 servings/day			P for trend*	P for trend**
Total cohort	N of death	HR	N of death	HR	95% CI	N of death	HR	95% CI	N of death	HR	95% CI	N of death	HR	95% CI		
Excluding those with smoking or diabetes	216	Ref.	11	0.65	0.33–1.28	16	0.78	0.43–1.41	35	1.22	0.81–1.83	19	2.12	1.26–3.57	0.206	0.002
Excluding those with death in 3 years	370	Ref.	21	0.66	0.39–1.11	34	1.0	0.67–1.5	71	1.28	0.95–1.71	37	1.67	1.15–2.45	0.077	0.002
Excluding those with BMI ≥30 kg/m2	398	Ref.	20	0.58	0.34–0.99	37	0.96	0.64–1.43	74	1.21	0.91–1.61	37	1.57	1.08–2.30	0.180	0.002
Excluding those with hypertension	204	Ref.	15	0.82	0.45–1.49	22	0.97	0.58–1.64	50	1.35	0.94–4.17	28	1.90	1.20–3.00	0.051	0.012
Excluding regular drinkers	279	Ref.	14	0.60	0.33–1.11	24	1.00	0.64–1.58	43	1.09	0.76–1.56	19	1.22	0.73–2.05	0.781	0.047

We referred to the age and the variables at cohort enrolment in this study.

HR was adjusted for categories of age, gender, education levels, smoking status, drinking status, physical activity, body mass index, hypertension, and diabetes.

*Trend starts from 0 to <0.5 serving/day.

**Trend starts from ≥0.5 to <1 serving/day.

## Discussion

SSB consumption was independently associated with pancreatic cancer in this Asian cohort. The risk remained when confounders such as smoking, diabetes, obesity, and hypertension were adjusted or excluded. The risk started at ≥1 servings/day for age < 40 years and ≥2 servings/day for all age groups. Moreover, a dose–response relationship was observed for cancer starting at 1 serving every other day. The increased risks remained even when all confounders, such as smoking, drinking, obesity, hypertension, and diabetes, were excluded or when death within the first 3 years of enrollment was excluded, implying the independent nature of the association.

With a large sample size, this is the first study to report the increased cancer risk for the entire Asian cohort, particularly among the younger population. The finding that young people have the highest risk of this highly fatal disease is of great concern. First, the risk started at ≥1 serving/day and not ≥2 servings/day as was noted for the remainder of the group, highlighting the vulnerability of the young population. Second, young people exhibited a higher prevalence and frequency of SSB intake than older people did, and their habitual SSB consumption typically started during their teenage years, making its weaning extremely difficult. Third, the young group also developed a taste for handmade bubble milk tea, which has high sugar content. This specialty drink was first invented in Taiwan. With a continuous increase in its popularity, this drink now occupies a large market share among sweetened beverages, both in Asia and globally (45).

From the perspective of Hill’s criteria of causality (46), the relationship between the increase in pancreatic cancer risk and SSB consumption observed in this study may not only be a chance association among the younger generation. First, the risk coincided with the highest consumers among all age groups. As reported in the United States, younger participants consumed three times more SSB in quantity than older participants (47). In the present study, participants aged <40 years exhibited a three times higher prevalence for each of the three different servings (≥0.5 to 1 serving/day: 17.2% vs. 4.5%; ≥1 to 2 servings/day: 15.4% vs. 6.1%; ≥ 2 servings/day: 9.3 vs. 3.5%; **Table 1**), implying that each young person on average consumed three times more than an older person (**Supplementary Figure 3**). Second, a dose–response relationship was observed within this age group and within the total cohort, with the highest risks observed among those who consumed the most drinks (2 servings/day or more). Men consumed more drinks and exhibited higher risks. Third, the increases remained even after most of the known pancreatic cancer risks, such as smoking, diabetes, drinking, hypertension, and obesity, were excluded, implying the independent nature of the relationship. Fourth, with pancreatic cancer having a 5-year survival rate of <10%, exclusion of people who died within the first 3 years of enrollment would have eliminated most pre-existing conditions. Fifth, SSB consumption is related to insulin release, which is known to be associated with pancreatic cancer (20–23, 29, 42). These factors pointed more favorably toward its causal association (46).

Similar to the increase in pancreatic cancer during the past 2 decades in the United States (1–4), Taiwan has experienced an approximately three-fold increase in pancreatic cancer incidence (5, 6). Smoking and drinking cannot sufficiently explain the increasing pancreatic cancer incidence because these behaviors have been highly prevalent, both in the United States and Taiwan, for decades before the increase in pancreatic cancer cases became evident. Moreover, in Taiwan, the number of both alcohol drinkers and adult smokers has declined in the past 10–20 years (48, 49). Between 1955 and 2016, the prevalence of current adult smokers in the United States also declined markedly (50). By contrast, the increase in pancreatic cancer incidence over the past 3 years has been accompanied by persisent high SSB consumption (51). The amount of SSB consumed has tended to increase gradually or remain stable worldwide, although the intake amount in the United States, Canada, the United Kingdom, and Australia has declined in the most recent decade. However, **Supplementary Table 3** showed that the temporal relationship between SSB intake and pancreatic cancer mortality was similar to the 10–20 years lagged effect of tobacco smoking or alcohol consumption on overall cancer mortality (52). Moreover, the sales figure of SSB in Taiwan has increased 8.9% annually from 2005 to 2019 (53). Bubble milk tea, an emerging popular drink, is worth mentioning as an addition to the existing sweetened beverages in the last 3–4 decades. The market has been growing since the launch of the product in the 1980s, and it is expected to double in the coming decade (45, 54). An aggravating factor is the reluctantance of Asians to use sugar substitutes, for soft drinks and for bubble milk tea, for fear of them being potential carcinogens. The extent to which bubble milk tea has exacerbated the cancer risk remains to be investigated.

The strengths of this study include its large sample size, a long follow-up period (median: 15 years), a cohort design rather than a case–control design, exclusion of participants who died within 3 years of enrollment, a series of sensitivity analyses conducted with risks found in both incidence and mortality, and high-quality cancer incidence data collected from the nationwide cancer registry (55, 56). A case–control study is subject to 1) recall bias because diseased individuals are reporting more exposure than their healthy counterparts, and 2) difficulty in replicating the results because the reference group is small and may not be sufficiently representative. The case–control case number ratio is often 2:1 or 4:1, but the ratio in our cohort study was approximately 500:500,000 or 1:1,000, with the reference group being much more representative.

The study also has several limitations. First, the study might be subject to selection bias because only people who could afford the membership fee were likely enrolled in this self-paid screening program. Different from other self-paid medical screening programs, the MJ clinics emphasize family-centered screening, with incentives to recruit more family members, namely, members of the extended family such as uncles, cousins, or grandparents, paid for by the head of the household. Therefore, MJ participants were from almost all levels of social classes, and selection bias, as commonly perceived, was minimized. With half a million participants, constituting nearly 3% of the Taiwan population, the socioeconomic status effect was further mitigated with the internal comparison study design analyzing relative risks in this study. Furthermore, our cohort exhibited a prevalence of risk factors, incidence, and mortality of cancer that is consistent with the values for the general Taiwan population (36, 39). Second, only data from the self-reported questionnaires from initial visits were used, and the dietary habits were subject to individual bias and changed with time. We also examined questionnaires from those who returned for a second visit and noted that the reported amount of SSB consumption was highly similar between the two visits. When the caloric contribution from sweetened beverages nears 15% of daily energy consumption, with 280 Kcal from 2 servings in an individual consuming 2,000 Kcal, some dietary replacement or modification may be adopted, leading to considerable nutritional implications for some individuals. However, we observed the increased pancreatic cancer risk, regardless of the variable amount of dietary modification. Nevertheless, randomized trials are required to ascertain the causal relationship between SSB and pancreatic cancer (16–38, 42, 43). Third, all sweetened drinks consumed in this cohort were assumed to contain real sugar and not sugar substitutes. As mentioned, sugar substitutes are viewed in Taiwan as potential carcinogens, and nearly all soft drinks consumed and 100% of handmade bubble milk tea do not contain these substitutes (47, 57). Regardless of the amount of substitutes, the drinks were associated with cancer risks. Fourth, although we adjusted for numerous variables in calculating the Cox model, some residual confounding factors could have been overlooked. Based on the literature, we believe that none of the residual risks would have a risk sufficiently high to affect our conclusion. Fifth, we studied the Asian population and results may not apply to non-Asians. Similar studies have been conducted worldwide, with some positive and negative results. With an increase in the pancreatic cancer rate in the United States, Taiwan, and elsewhere, future research could verify our results with a larger adult sample.

Our results indicate that SSB intake was independently associated with pancreatic cancer in this Asian cohort, with highest risks among young people (age <40 years). Starting from one drink a day, a dose–response relationship was observed between the amount of SSB intake and pancreatic cancer risk. The risk was compounded by the increasing popularity of bubble milk tea. Considerable effort should be devoted to encourage modification of SSB consumption behavior.

## Data Availability Statement

The raw data supporting the conclusions of this article will be made available by the authors, without undue reservation.

## Ethics Statement

The studies involving human participants were reviewed and approved by the China Medical University & Hospital Research Ethics Committee. The patients/participants provided their written informed consent to participate in this study.

## Author Contributions

Study concept and design: CPW and CHC. Analysis and interpretation of data: MKT, CHL, and CW. Drafting of the manuscript: CPW and CHC. Critical revision of the manuscript for important intellectual content: CPW, CHC, RTL, CW, MKT, and CHL. Technical or material support; study supervision: CPW, CHC, CYH, XW, and TWDC. All authors listed have made a substantial, direct, and intellectual contribution to the work and approved it for publication.

## Funding

This study is supported in part by the Taiwan Ministry of Health and Welfare Clinical Trial Center (MOHW109-TDU-B-212-114004), the MOST Clinical Trial Consortium for Stroke (MOST 109-2321-B-039-002), the China Medical University Hospital (DMR-109-231), and the Tseng-Lien Lin Foundation, Taichung, Taiwan.

## Conflict of Interest

The authors declare that the research was conducted in the absence of any commercial or financial relationships that could be construed as a potential conflict of interest.

## Publisher’s Note

All claims expressed in this article are solely those of the authors and do not necessarily represent those of their affiliated organizations, or those of the publisher, the editors and the reviewers. Any product that may be evaluated in this article, or claim that may be made by its manufacturer, is not guaranteed or endorsed by the publisher.

## Abbreviations

SSB, sugar-sweetened beverages; MET, metabolic equivalent task; HR, hazard ration; CI, confidence interval; BMI, body mass index; SES, socioeconomic status.
