# Supplementary material for: “Sugar-Sweetened Beverages” Is an Independent Risk From Pancreatic Cancer: Based on Half a Million Asian Cohort Followed for 25 Years
Source: Front Oncol. 2022 Apr 7;12:835901. doi: 10.3389/fonc.2022.835901 (PMC9022008; doi:10.3389/fonc.2022.835901)
Supplement: Supplementary file 1 [file DataSheet_1.docx]

Supplementary Table 1. Incidence risks of pancreatic cancer by levels of consumed sugar-sweetened beverages

|  | 0 - < 0.5  serving/day | | ≥ 0.5 - < 1  serving/day | | | ≥ 1- < 2  servings/day | | | ≥ 1  serving/day | | | ≥ 2  servings/day | | |  |  |
| --- | --- | --- | --- | --- | --- | --- | --- | --- | --- | --- | --- | --- | --- | --- | --- | --- |
|  | N | HR | N | HR | 95% CI | N | HR | 95% CI | N | HR | 95% CI | N | HR | 95% CI | P for trend* | P for trend** |
| Total cohort | 386 | Ref. | 26 | 0.67 | 0.42-1.07 | 38 | 0.88 | 0.59-1.30 | 77 | 1.15 | 0.87-1.52 | 39 | 1.55 | 1.08-2.23 | 0.233 | 0.002 |
| Age 20-39 at enrollment | 32 | Ref. | 8 | 1.27 | 0.57-2.81 | 6 | 0.68 | 0.24-1.96 | 18 | 1.53 | 0.82-2.86 | 12 | 2.67 | 1.34-5.33 | 0.047 | 0.086 |

We referred to the age and the variables at cohort enrolment in this study

For total cohort, HR was adjusted for categories of age, gender, education levels, smoking status, drinking status, physical activity, body mass index, hypertension, and diabetes

For age-classified models, HR was adjusted for gender, education levels, smoking status, drinking status, physical activity, body mass index, hypertension, and diabetes

*Trend starts from 0 - < 0.5 serving/day

**Trend starts from ≥ 0.5 - <1 serving/day

Supplementary Table 2. Sensitivity analyses for the mortality risks of pancreatic cancer by levels of consumed sugar-sweetened beverages in age group 20-39 at enrollment.

|  | 0 - < 0.5  serving/day | | ≥ 0.5 - < 1  serving/day | | | ≥ 1 - < 2  servings/day | | | ≥ 1  serving/day | | | ≥ 2  servings/day | | | P for trend* | P for  trend** |
| --- | --- | --- | --- | --- | --- | --- | --- | --- | --- | --- | --- | --- | --- | --- | --- | --- |
| Age 20-39 at enrollment | N of death | HR | N of death | HR | 95% CI | N of death | HR | 95% CI | N of death | HR | 95% CI | N of death | HR | 95% CI |  |  |
| Excluding those with smoking or diabetes | 15 | Ref. | 1 | 0.35 | 0.05-2.69 | 2 | 0.40 | 0.05-3.04 | 10 | 2.19 | 0.93-5.19 | 8 | 5.11 | 2.09-12.53 | 0.010 | 0.003 |
| Excluding those with death in 3 years | 25 | Ref. | 4 | 0.94 | 0.32-2.76 | 7 | 1.57 | 0.63-3.92 | 17 | 2.31 | 1.19-4.47 | 10 | 3.23 | 1.50-6.97 | 0.005 | 0.029 |
| Excluding those with BMI ≥ 30 kg/m^2^ | 23 | Ref. | 2 | 0.55 | 0.13-2.36 | 5 | 1.17 | 0.39-3.46 | 14 | 2.14 | 1.04-4.42 | 9 | 3.47 | 1.53-7.85 | 0.012 | 0.009 |
| Excluding those with hypertension | 23 | Ref. | 3 | 0.89 | 0.26-3.03 | 4 | 0.94 | 0.28-3.23 | 12 | 1.95 | 0.91-4.17 | 8 | 3.27 | 1.40-7.67 | 0.030 | 0.031 |
| Excluding regular drinkers | 16 | Ref. | 2 | 0.61 | 0.14-2.69 | 4 | 1.01 | 0.29-3.56 | 43 | 1.09 | 0.76-1.56 | 7 | 3.32 | 1.30-8.52 | 0.045 | 0.016 |

We referred to the age and the variables at cohort enrolment in this study

HR was adjusted for gender, education levels, smoking status, drinking status, physical activity, body mass index, hypertension, and diabetes

* Trend starts from 0 - < 0.5 serving/day

** Trend starts from ≥ 0.5 - <1 serving/day

Supplementary Table 3. The development of pancreatic cancer incidence and mortality in each age group at enrollment.

|  | Age at enrollment  (mean ± standard deviation) | Age for pancreatic cancer incidence  (mean ± standard deviation) | Age for pancreatic cancer mortality  (mean ± standard deviation) |
| --- | --- | --- | --- |
| Total cohort | 39.9 ± 13.2 | 65.6 ± 13.0 | 68.1 ± 12.0 |
| 20-39 years | 30.6 ± 4.8 | 43.1 ± 6.9 | 46.5 ± 5.5 |
| 40-59 years | 48.5 ± 5.9 | 60.4 ± 7.4 | 62.0 ± 6.9 |
| ≥ 60 years | 66.5 ± 5.6 | 75.4 ± 7.0 | 76.8 ± 6.7 |

Age was attributed to the age of cancer diagnosis.

Age was attributed to the age of cancer diagnosis.

Supplementary Figure 3. Ratio of any amount of sugar-sweetened beverages consumption between different age groups, for Taiwan and US.

Sugar-sweetened beverages per day of US data was from (46)
